# Fundamental and emerging insights into innate and adaptive immunity in inflammatory bowel diseases

**DOI:** 10.3389/fimmu.2025.1665530

**Published:** 2025-10-21

**Authors:** Giovanni Monteleone, Carlo Calisi, Silvia Salvatori, Irene Marafini

**Affiliations:** ^1^ Gastroenterology Unit, Azienda Ospedaliera Policlinico Tor Vergata, Rome, Italy; ^2^ Department of Systems Medicine, University of “Tor Vergata”, Rome, Italy

**Keywords:** ulcerative colitis, Crohn’s disease, mucosal immunology, cytokines, IBD

## Abstract

Inflammatory bowel diseases (IBD) are chronic and disabling disorders of the gastrointestinal tract of unknown aetiology, in which the pathologic process is triggered by multiple environmental and genetic factors that activate an excessive innate and adaptive immune response against luminal antigens. In recent years, great progress has been made in the identification of factors/mechanisms underlying the amplification of the key immune steps in IBD tissue, and this has facilitated the development of several immune-related biotherapeutic compounds that have largely improved the management of the more severe forms of IBD. However, nearly half of these patients are refractory or intolerant to novel immunotherapeutics, indicating the need for further characterization of the IBD-associated detrimental immune response to develop new therapeutics. In this article, we review the available evidence about the contribution of innate and adaptive immune cells in the development of intestinal tissue damage. We also discuss the more recent findings in the field of IBD-associated immunity, which might help identify novel pathways to be manipulated for therapeutic purposes.

## Introduction

Inflammatory bowel diseases (IBD) are immune-mediated diseases characterized by a chronic, relapsing intestinal inflammation with two major subtypes, ulcerative colitis (UC) and Crohn’s disease (CD). In UC, the inflammation arises in the mucosal layer of the rectum and can extend proximally and continuously to the whole colon. In the severe cases of UC, the inflammation can involve the submucosal compartment. In CD, the inflammation is transmural and segmental and can involve any part of the gastrointestinal tract (1). The natural history of IBD can be complicated by the development of local complications (e.g., abscesses, toxic megacolon, colon cancer) and/or extraintestinal manifestations, mainly involving the joints, eyes, and skin (2). Paradoxical manifestations (i.e., cutaneous and/or articular lesions) can also occur in some IBD patients following the use of biologics; these manifestations are often reversible after the discontinuation of the implicated drug (3).

IBD etiology remains unknown, but accumulating evidence supports the hypothesis that these are multifactorial disorders, with contributions from host genetics, environment, and intestinal microbiota, and characterized by the activation of innate and adaptive immune responses, excessive production of inflammatory cytokines, and various degrees of tissue destruction (4).

Dissecting the molecular events that amplify and sustain the IBD-associated pathological inflammation has facilitated the development of several biologics and small molecules, which have improved the management of these disorders. Most of these drugs target inflammatory cytokines and cytokine-activated intracellular kinases, or interfere with mechanisms involved in the recruitment of immune cells to the inflamed gut, further supporting the notion that CD and UC are immunologically-mediated diseases (5). However, a considerable proportion of IBD patients are unresponsive to biologics and/or small molecules, highlighting the necessity of further studies to advance our understanding of the mechanisms promoting the IBD-associated mucosal inflammation and find effective treatments.

In this article, we review the available evidence about the contribution of innate and adaptive immune cells in the development of intestinal tissue damage with particular regard to effector cells. We also discuss the more recent findings in the field of IBD-associated immunity, which might help identify novel pathways to be manipulated for therapeutic purposes.

## Genetic and experimental studies support the pathogenic role of innate immunity dysfunction in IBD

Several pieces of evidence indicate that both CD and UC have a genetic basis. For instance, it has long been known that patients with IBD have a positive family history, and the concordance rate for IBD is significantly higher in monozygotic (identical) twins compared to dizygotic (fraternal) twins (6). Additionally, more than 240 susceptibility genes and single-nucleotide polymorphisms (SNPs) have been identified by genome-wide association studies (GWAS) in IBD, and many of these gene variants are involved in the innate response to microbes as well as in the regulation of adaptive immunity (7–9).

Nearly one-third of CD patients bear one or more SNPs situated in or close to the leucine-rich repeat ligand-binding domain of nucleotide-binding oligomerization domain-containing 2 (NOD2), also known as caspase recruitment domain protein 15 (CARD15). Three coding SNPs within the gene, designated SNP8, SNP12, and SNP13 have been associated with CD in Caucasians but not in Asians (10). Specifically, SNP8 and SNP12 cause amino acid substitutions in the leucine-rich region (LRR), namely C14772T (Arg702Trp) and G25386C (Gly908Arg) (11). SNP13 is a C-insertion 32629insC (1007insC), which leads to a frameshift that causes a truncated protein missing the fInal 33 amino acids (10). Although initial studies documented no association between NOD2 SNPs and UC, a study conducted in a Portuguese population of UC patients showed that NOD2 variants correlate with a more aggressive course of the disease (12). Additionally, a study conducted in Punjab (India) showed that the 3 disease susceptibility variants were rare, but identified two additional SNPs (SNP5, 268 Pro / Ser and rs2067085, 178 Ser / Ser). The frequency of SNP5 was higher in both UC and CD patients than in controls (12%), and SNP5 carriers had elevated risks for UC (13).

NOD2, an intracellular sensor for the muramyl dipeptide of peptidoglycan, is expressed in many immune cells as well as in Paneth cells, and positively regulates the production of α-defensin (14). Indeed, CD patients bearing NOD2 SNPs have a diminished activation of nuclear factor kappa-light-chain-enhancer of activated B cells (NF-kB) and decreased production of defensins (15). NOD2-deficient mice develop granulomatous inflammation of the ileum, characterized by a marked enlargement of the Peyer’s patches and mesenteric lymph nodes and increased production of T helper type (Th1)-related cytokines following inoculation with Helicobacter hepaticus, an opportunistic pathogenic bacterium (16). Notably, restoring the crypt antimicrobial function of NOD2-deficient mice by transgenic expression of α-defensin in Paneth cells rescues the Th1 inflammatory phenotype (16). The mechanisms by which NOD2 SNPs predispose to CD are multiple, probably reflecting the ability of the protein to regulate various functions in different cell types, including the production of interleukin (IL)-12 in response to Toll like receptor (TLR)2 (17), and of IL-10 through a pathway mediated by the association of NOD2 with active p38 mitogen-activated protein kinase, and the transcription factor heterogeneous nuclear ribonucleoprotein A1 (18).

**Figure 1:**
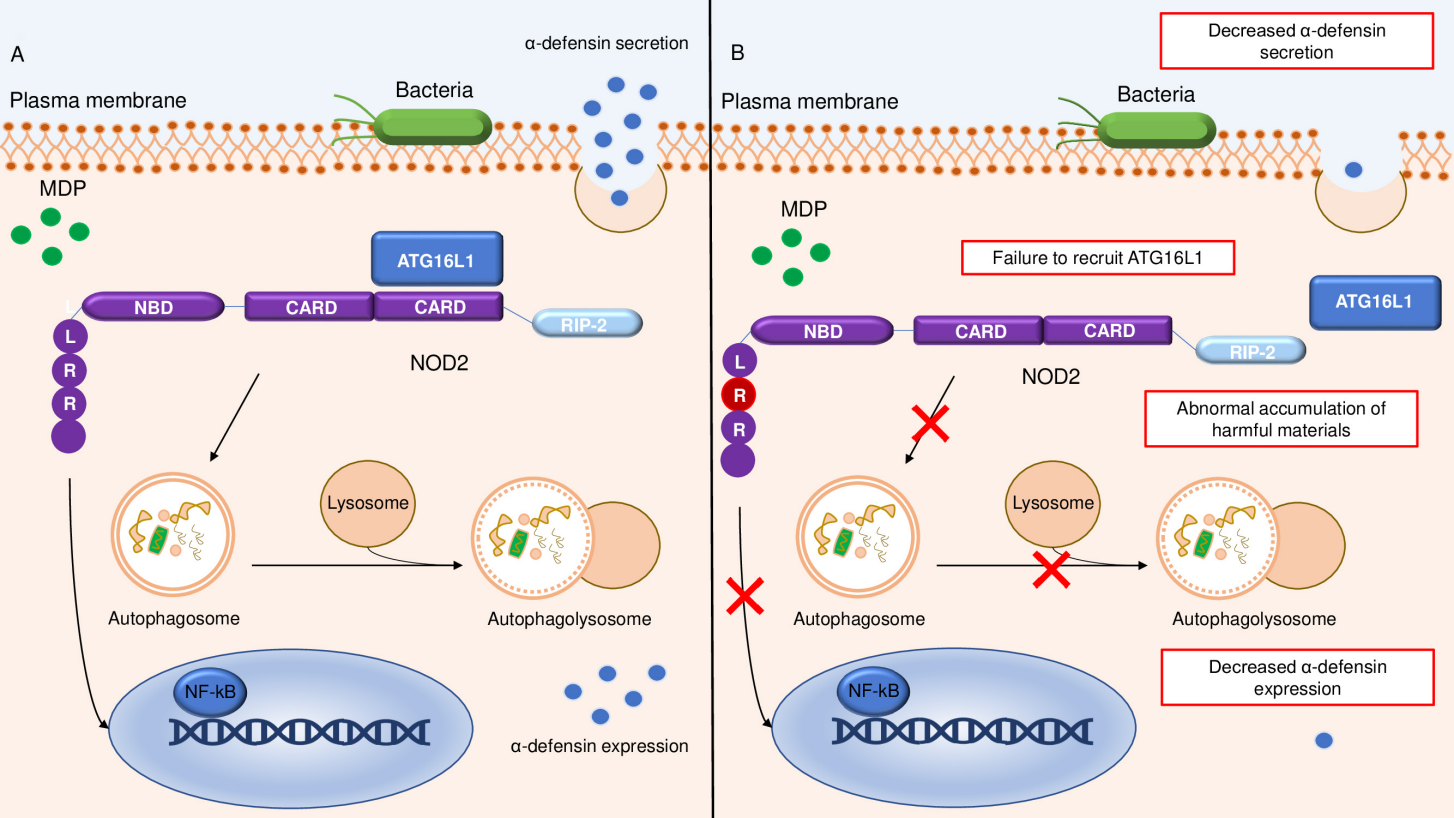
Schematic representation of the relationship between NOD2 and ATG16L1 in the autophagy pathway. **(A)** shows a normal autophagy response with recruitment of ATG16L1 to the plasma membrane by NOD2, followed by autophagosome and autophagolysosome formation and conserved α-defensine expression and secretion. **(B)** Altered autophagy pathway due to SNPs in leucine-rich repeat ligand-binding domain of NOD2, resulting in defective ATG16L1 recruitment, reduced autophagosome and autophagolysosome formation, and diminished intracellular bacterial killing with accumulation of harmful materials and decreased α-defensine expression and secretion. NOD2, nucleotide-binding oligomerization domain-containing 2; LRR, Leucine-Rich Repeat; NBD, nucleotide binding domain; RIP2, Receptor-Interacting Protein 2; MDP, Muramyl dipeptide; NF-kB, Nuclear Factor kappa-light-chain-enhancer of activated B cells.

NOD2 also controls autophagy, a process that is responsible for the degradation and elimination of damaged organelles or long-lived proteins and bacterial clearance (19). Specifically, this process relies on the ability of NOD2 to recruit the autophagy protein ATG16L1 to the plasma membrane at the site of bacterial entry (20) (**Figure 1**). Defects in autophagy can also be secondary to a SNP in the ATG16L1 gene (rs2241880; leading to a T300A conversion), which is associated with risk for developing CD (21, 22). Impairment of the autophagy process due to the rs2241880 variant leads to an abnormal cellular accumulation of harmful materials (23) (**Figure 1**). In both CD subjects and mice with ATG16L1T300A, cigarette smoking, a major CD environmental risk factor (24–26), triggers Paneth cell defects, including enhanced apoptosis and metabolic dysregulation, due to a selective downregulation of the proliferator-activated receptor-gamma (PPARγ) pathway (27). ATG16L1 can specifically interact with murine norovirus (MNV), and there is evidence that hypomorphic ATG16L1 mice infected by MNV CR6 exhibit morphological and granule-packaging abnormalities in Paneth cells (28). Furthermore, ATG16L1 prevents tumor necrosis factor (TNF)-α-mediated Paneth cell necroptosis by maintaining mitochondrial homeostasis (29). ATG16L1 is also expressed in immune cells, such as lymphocytes and antigen-presenting cells. Macrophages isolated from ATG16L1-deficient mice produce high amounts of inflammatory cytokines in response to bacterial challenges and are more susceptible to experimental colitis (30). Another gene linked to CD is immunity-related GTPase M (IRGM), a negative regulator of the NLRP3 inflammasome activation and pro-inflammatory responses to microbial stimuli. Consequently, defects in IRGM lead to enhanced pyroptosis and gut inflammation in mice (31). Overall, these observations indicate that various SNPs can alter the ability of innate immune cells to recognize and respond to intracellular bacteria, thereby leading to a pathological process that eventually causes gut mucosal damage. Support for this notion comes from the demonstration that some congenital disorders of innate immunity (particularly of phagocyte function) are associated with a CD-like noninfectious bowel inflammation (32). Similarly, some individuals suffering from various neutropenias (i.e., Hermansky-Pudlak syndrome, chronic granulomatous disease, leukocyte adhesion deficiency-1, Chediak-Higashi syndrome, and glycogen storage disease type 1b) can develop CD-like intestinal inflammation (33).

## Excessive innate immune responses against luminal microorganisms lead to intestinal inflammation

In IBD, intestinal microbiota provides an abundant source of immunostimulatory molecules that trigger immune responses. Consistent with this is the demonstration that the inflamed gut of IBD patients contains a high number of antigen-presenting cells that display an activated phenotype, express high levels of TLRs, and respond to microbial components by producing huge amounts of inflammatory cytokines, such as IL-1β, IL-6, IL-18, and TNF, the expression of which correlates with the severity of inflammation (34). Such cytokines are mainly produced by CD14+ myeloid cells (35), suggesting that the major contribution to the inflammatory cytokine overproduction is given by monocytes recently recruited from the blood. Antigen-presenting cells also synthesize the heterodimeric IL-12 and IL-23, which share the p40 subunit, and Epstein-Barr virus-induced gene 3 (EBI3)-related cytokines such as IL-27 and IL-35 that, as discussed below, control Th cell polarization (36, 37). Microbial components signal through TLRs and activate various intracellular pathways (e.g., MAP kinases) (38). Indeed, IBD lamina propria immune cells exhibit increased NF-kB activity, and blockade of this transcription factor reduces the production of IL-1β, IL-6, and TNF and attenuates disease in mouse models of intestinal inflammation (39). However, in IBD, NF-kB is also expressed by epithelial cells, where it has beneficial effects on the maintenance of gut homeostasis (40).

Both IL-1β and IL-18 are produced as pro-cytokines and can then be cleaved into a mature/active form by caspase 1. In this process, which is induced by harmful stimuli, such as invading pathogens, dead cells, or environmental irritants, various members of the LRR-containing proteins (NLR) family can associate with the NLR adaptor protein, apoptosis-associated speck-like protein containing a CARD domain (ASC/PYCARD), to recruit procaspase 1 and favor the processing of procaspase 1 into active caspase 1. This complex is referred to as inflammasome, the activation of which can be induced by two distinct mechanisms. In the non-canonical pathway, intracellular LPS promotes the activation of Caspases-11/4/5, which induces the cleavage and activation of Gasdermin (GSDM)-D, leading to cell swelling and pyroptosis. In this process, caspase-11 also activates pannexin-1, a protein channel that releases ATP from the cell. The extracellular ATP favors the opening of a pore that enhances potassium ion efflux, thus driving NLRP3 inflammasome assembly and secretion of the active forms of IL-1β and IL-18 by caspase-1. In the alternative pathway, activation of the inflammasome is triggered in myeloid cells by extracellular LPS, and is marked by the activation of caspase-8 and subsequently NLRP3, and does not induce pyroptosis. Through an evaluation of seven Gene Expression Omnibus datasets, Gao and colleagues assessed the correlation between hub gene expression and anti-TNF therapy outcomes in IBD patients. Several genes involved in the inflammasome activation and pyroptosis (i.e., caspases 1,5, GSDM-D, AIM2, and NLRP3) predicted the response to anti−TNF therapy, and non-responders to TNF blockers exhibited elevated AIM2 protein expression (41). The driving role of the inflammasome in the early development of gut inflammation is supported by studies in mice deficient for NLRP3, ASC, or caspase 1. Such animals produce reduced amounts of IL-1β and TNF and are protected from acute, but not chronic, experimental colitis (42). Guanylate binding protein 5 (GBP5), an interferon-stimulated gene that is highly up-regulated in IBD, enhances the expression of pro-inflammatory cytokines in mononuclear cells as a result of its ability to promote NLRP3 inflammasome activation through a not yet defined mechanism (43–46). Strikingly, GBP5-deficient mice are resistant to dextran sulfate sodium (DSS)-induced colitis, further corroborating the role of GBP5 in gut inflammation (47). However, the inflammatory role of GBP5 is not restricted to the control of inflammasome, as GBP5 can trigger the expression of numerous pro-inflammatory cytokines and chemokines through an inflammasome-independent mechanism, which is mediated by STAT1 and leads to innate lymphoid cell (ILC) proliferation and, eventually, intestinal inflammation in a DSS mouse model (48).

## Factors that amplify the innate immune response in IBD

**Figure 2:**
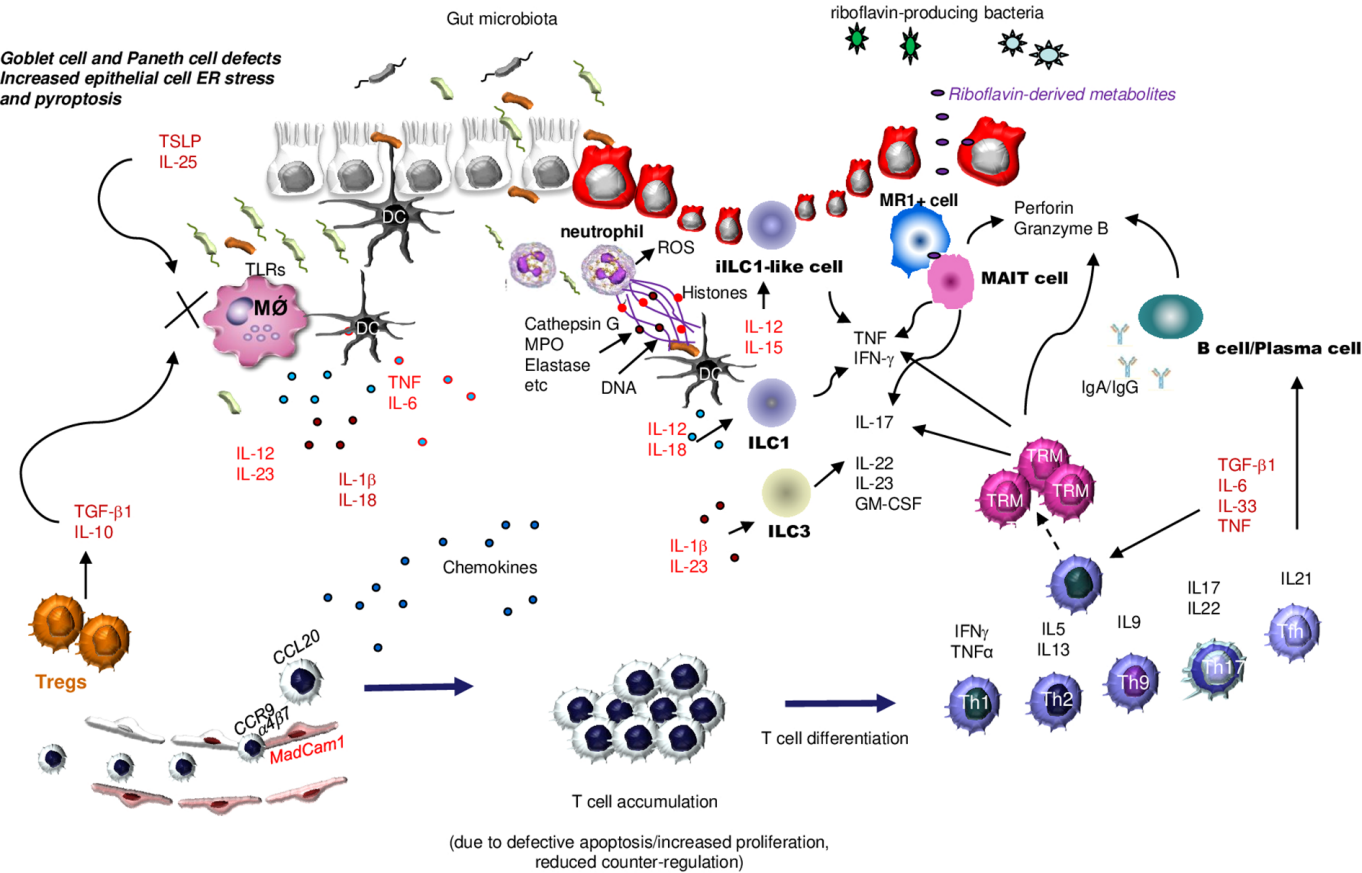
Schematic view of the main innate and adaptive immune cells involved in IBD pathogenesis. As a result of multiple epithelial cell defects that enhance translocation of luminal microorganisms into the lamina propria, antigen-presenting cells, such as dendritic cells (DC) and macrophages (MǾ produce huge amounts of various cytokines. Such a production is amplified by diminished production of epithelial cell- and regulatory T cells (Tregs)-derived counter-regulatory molecules [i.e., Thymic stromal lymphopoietin (TSLP) and interleukin (IL)-25]. The inflamed gut of IBD is also heavily infiltrated with additional innate immune cells, such as neutrophils that secrete reactive oxygen species (ROS), chemokines, and neutrophil extracellular traps (NETs), various subsets of cytokine-producing innate lymphoid cells (ILC), and mucosal-associated invariant T (MAIT) cells, the latter being activated by riboflavin-derived metabolites. T cells are recruited in the gut in response to the action of locally-produced chemokines, and this process is facilitated by interaction between integrins expressed on the lymphocyte surface (e.g., a4b7) and adhesion molecules expressed by endothelial cells (e.g., MadCam1). T cells accumulate in the lamina propria as a result of additional mechanisms, and then differentiate into various subsets, which contribute to enhancing the amount of inflammatory cytokines and to promoting IgG- and Granzyme-B-secreting plasma cell differentiation. Under the stimulus of specific effector cytokines, the activated T cells can become memory T cells, another subset of cytokine- and perforin and Granzyme B-producing T cells. ER, Endoplasmic Reticulum; TSLP, Thymic Stromal Lymphopoietin; TLR, Toll-like receptor; DC, Dendridic cell; ROS, Reactive Oxygen Species; MPO, Myeloperoxidase; ILC1, innate lymphoid cells type 1; ILC3, innate lymphoid cells type 3; TRM, Tissue-Resident Memory T cells; MR1+ cell, major histocompatibility complex (MHC) class I-related molecule 1 (MR1)-restricted T cell; MAIT cell, Mucosal-associated invariant T cells; TNF, Tumor Necrosis Factor; TGFβ1, Transforming Growth Factor β1; IL-1β, interleukin-; IL-5, interleukin-5; IL-6, interleukin-6; IL-9, interleukin-9; IL-10, interleukin-10; IL-12, interleukin-12; IL-13, interleukin-13; IL-15, interleukin-15; IL-17, Interleukin-17; IL-18, interleukin-18; IL-22, interleukin-22; IL-23, interleukin-23; IL-25, interleukin-25; IL-33, interleukin-33; IFNγ, interferon γ; TH1, T helper 1 cells; TH2, T helper 2 cells, TH9, T helper 9 cells; TH17, T helper 17 cells; Tregs, regulatory T cells; Tfh, T follicular helper cells; CCL20, C-C motif chemokine ligand 20; CCR9; MadCam1, Mucosal Addressin Cell Adhesion Molecule 1; GM-CSF, Granulocyte-Macrophage Colony-Stimulating Factor.

Additional factors/mechanisms contribute to sustaining the excessive innate immune response in IBD. For example, in the inflamed mucosa of IBD patients, there is over-expression of OTUD5, an enzyme that cleaves ubiquitin linkages, thus resulting in enhanced protein stability and altered signal transduction (49). OTUD5 protein is highly expressed in IBD mucosa, mainly by epithelial cells and myeloid cells, and knockdown of OTUD5 with a specific antisense oligonucleotide reduces TNF production (50). IBD mucosal cells express reduced levels of SIRT1, a class III NAD+-dependent deacetylase, which inhibits the expression of various proteins involved in the control of immune-inflammatory pathways, such as STAT3, Smad7, and NF-κB. Notably, *in vitro* treatment of IBD mucosal immune cells with Cay10591, a specific SIRT1 activator, reduces NF-κB activation and inhibits the production of inflammatory cytokines, as well as Cay10591 prevents and cures IBD-like colitis in mice (51). Intestinal antigen-presenting cells in IBD patients exhibit a defective expression of programmed death (PD) ligand 1 (PDL1) (52), which is known to engage PD1 on T cells thereby promoting the function of T-regulatory cells (Tregs), a class of T cells expressing CD25 and the transcription factor Foxp3 and known to maintain immune tolerance (53). The promoter regions of human and mouse Pdcdl1 (PDL1) and Pdcdl2 (PDL2) genes contain multiple putative binding sites for Smad3, a transcription factor that is activated by transforming growth factor (TGF)-β1 (54), the activity of which is reduced in IBD mucosa due to high levels of Smad7, an intracellular inhibitor of TGF-β1/Smad3 signaling (55, 56). Deletion of Smad7 in dendritic cells (DC) enhances TGF-β1 responsiveness and increases PDL1/2-PD1 signaling and Treg differentiation, thereby protecting mice from intestinal inflammation (54). Moreover, several molecules that counter-regulate myeloid cell function and innate immune responses (e.g., Thymic stromal lymphopoietin and IL-25) are reduced in IBD tissue (57, 58) (**Figure 2**). Another cytokine involved in the activation of inhibitory pathways in the gut is IL-10. Initial studies in IL-10-deficient mice showed the role of IL-10 in suppressing antigen-presenting cell function, given that mutants exhibited excessive bacteria-induced IL-12 production and developed enterocolitis (59). Consistently, germ-line mutations causing loss of IL-10 signaling can be associated with a very early-onset IBD (60).

In the inflamed gut of IBD patients, there is also a reduced expression of aryl hydrocarbon receptor (AHR) (61), the activation of which in DC leads to a reduced expression of activation markers (i.e. CD80, CD83, and CD86) and diminished production of inflammatory cytokines (i.e., IL-1β, IL-23, and IL-12). Additionally, AHR activation facilitates the differentiation of regulatory DC and Tregs, thus limiting experimental colitis in mice (62–64). In IBD, DC also exhibit an altered activation state, characterized by increased production of pro-inflammatory cytokines such as IL-12 and IL-6, with reduced IL-10 expression. This shift promotes pathogenic Th1 and Th17 responses and contributes to the breakdown of mucosal tolerance. DC also display enhanced expression of co-stimulatory molecules, further amplifying the ongoing T cell activation. These changes support the hypothesis that DC dysregulation plays a central role in initiating and sustaining intestinal inflammation (65).

Altogether, these observations indicate the existence of a multitude of factors and mechanisms that either simultaneously or sequentially contribute to expanding the local innate immune response against luminal microorganisms, thereby resulting in excessive production of inflammatory cytokines.

## The role of neutrophils in IBD

The active phases of IBD are characterized by a mucosal accumulation of neutrophils, which correlates with the severity of the disease, while the resolution of neutrophil mucosal infiltration is associated with improved long-term clinical outcomes (i.e. use of corticosteroids, hospitalization) (66–68). The fecal concentration of calprotectin, a calcium- and zinc-binding protein mainly produced by neutrophils, is useful in the diagnosis and management of IBD and helps predict disease course (69).

Neutrophils are recruited from the bloodstream as a result of signals activated by molecules largely produced by epithelial cells (e.g., IL-8, IL-6, IL-33, CXCL5, CXCL7, CXCL10, and CCL20, leukotriene B4, and hepoxilin A3, and matrix metalloproteinases) (70). In contrast to blood neutrophils that have a very short half-life (8–12 hours), mucosal neutrophils can survive for 1–4 days. Moreover, in the inflamed gut of IBD patients, neutrophils exhibit defective apoptosis, a process that has been linked to the ability of a multitude of cytokines, hypoxia, extracellular acidosis, and bacterial products (e.g., LPS, lipoproteins, peptidoglycans, and lipoteichoic acid) to prolong neutrophil survival (71, 72). Resolution of mucosal inflammation is mediated in part by members of the superfamily of lipidic specialized proresolving mediators (SPMs), a class of lipids biosynthesized from omega-6 and omega-3 polyunsaturated fatty acids. SPMs target distinct G protein-coupled receptors (GPCRs), thus stopping neutrophil transmigration and triggering their apoptosis (73, 74). Through a transcriptional analysis of mucosal samples, Trilleaud and colleagues showed that ChemR23, a GPCR targeted by resolvin E1, was highly expressed in the inflamed gut of IBD patients unresponsive to biologics (i.e. TNFα blockers or vedolizumab) and associated with significant mucosal neutrophil accumulation. Moreover, treatment of colitic mice with an anti-ChemR23 antibody promoted the transition of pro-inflammatory macrophages to an anti-inflammatory phenotype, thereby leading to neutrophil apoptosis and clearance (75).

Within the gut mucosa, neutrophils secrete a variety of proteolytic enzymes and reactive oxygen species (ROS), which alter the epithelial barrier integrity, thereby facilitating their transepithelial migration and the formation of characteristic pathological lesions (i.e., cryptitis, crypt abscesses) (76). A recent study reported that, in IBD patients as well as in patients with chronic granulomatous disease and mice with experimental colitis, neutrophils express the NADPH oxidase DUOX2, which stimulates H_2_O_2_ production. Notably, DUOX2 enhances and perpetuates intestinal inflammation, increases tissue damage, and delays restitution, raising the possibility that DUOX2 may be an emerging therapeutic target to attenuate gut inflammation (77). A further contribution of neutrophils to IBD-associated inflammation relies on their ability to secrete several chemokines and cytokines (e.g., CXCL1, CXCL2, and CXCL5, IL-8, IL-1β, and IL-17) that promote the recruitment of other immune cells, such as monocytes, T cells, and NK cells (78). In this context, it is noteworthy that neutrophils can respond to microbial components by secreting neutrophil extracellular traps (NETs), an extracellular network composed of DNA, histones, and anti-microbial inflammatory proteins and proteases, including neutrophil elastase and myeloperoxidase, that traps and neutralizes pathogens, thereby preventing their further spread (79) (**Figure 2**).

NET formation (the process is also termed NETosis) is controlled by various factors, including ROS and transcription factors, which are triggered by microorganisms and several cytokines. Citrullination of histones H1, H3, and H4 by the enzyme peptidyl arginine deiminase 4 (PAD4) is crucial in the decondensation and expulsion of nuclear chromatin (80). The formation of NETs is increased in IBD mucosa (81) and correlates with disease activity (82). Notably, serum samples of IBD patients can enhance the formation of NETs in blood neutrophils of healthy individuals (83), suggesting that, in IBD, NETs can be induced by locally secreted factors. This hypothesis is supported by the demonstration that the formation of NETs can be stimulated by IL-1β through an autophagy-dependent mechanism (84). Indirect evidence also supports the hypothesis that the abundance of NETs in IBD mucosa reflects their defective clearance, primarily due to a reduction in the activity of deoxyribonuclease I (DNase I) (85).

A large body of evidence supports the pathogenic role of NETs in IBD. For instance, NETs can trigger epithelial cell apoptosis, thereby perturbing the intestinal barrier integrity and, hence, amplifying the ongoing inflammation in DSS- or 2,4,6-trinitrobenzene sulfonic acid (TNBS)-induced models of colitis. Treatment of colitic mice with intravenous DNase I, an enzyme that dissolves the web-like DNA filaments of NETs, restores the mucosal barrier integrity and attenuates intestinal inflammation (86). Consistently, PAD4-deficient mice are less susceptible to DSS-colitis (87).

We also showed that culturing UC lamina propria mononuclear cells (LPMCs) with NETs resulted in enhanced production of TNF-α and IL-1β, and inhibition of NET release attenuated DSS-colitis in mice (81). Moreover, NET formation was reduced in colons of UC patients who responded to treatment with TNF antagonists (81), further supporting the notion that NET generation is driven by cytokines produced within the inflammatory environment associated with the disease. NETs can adhere to the surface of vascular endothelial cells and promote apoptosis, a phenomenon that was linked mainly to the action of NET-related proteins (88). Finally, the therapeutic effect of various compounds in colitic mice has been associated with an inhibitory effect on NET formation (89, 90).

NETs could also act as a powerful fibrogenetic stimulus in CD, as in the inflamed ileum of CD patients, they colocalize with activated fibroblasts and stimulate fibroblasts to upregulate pro-fibrotic genes and increase collagen production. Moreover, mice with PAD4-deficient neutrophils exhibit a significant reduction in collagen content following repeat DSS exposure (91). Additional studies have linked NETs to the increased thrombotic tendency in IBD, due to the promoting effects of NETs on primary and secondary hemostasis (83, 92). However, a study published by Leppkes and colleagues showed that neutrophils induce PAD4-dependent immunothrombosis in UC, and NET-associated immunothrombi prevent rectal bleeding in DSS-colitis. Indeed, loss of PAD4 was associated with a failure to remodel blood clots on the mucosal surface, delayed colonic wound healing, and worsening of the ongoing colitis with a failure to control rectal bleeding (93).

Taken together these findings suggest that an unrestrained translocation of microbial antigens to the lamina propria induces a massive recruitment of neutrophils, which contribute to the mucosal damage through a variety of mechanisms (i.e., impairment of epithelial barrier function, mucosal injury through oxidative and proteolytic damage, and the amplification of the ongoing inflammatory reaction through the release of inflammatory mediators).

## The role of eosinophils in IBD

Eosinophils are increasingly recognized as active players in the pathogenesis of IBD (94, 95), as these cells could contribute to mucosal damage through the release of cytotoxic granules, cytokines (e.g., IL−5, IL−13), and ROS (95, 96). In IBD tissue, IL-5 receptor-α subunit expression on eosinophils is markedly increased and correlates with disease activity and endoscopic severity, especially in CD (95). Moreover, there is evidence that eosinophil accumulation is predictive of poor response to therapy and increased risk of relapse (97). Animal models show that eosinophil depletion reduces intestinal inflammation (98). Altogether, eosinophils emerge as both biomarkers and potential therapeutic targets in IBD (97).

## The role of innate lymphoid cells in the control of IBD-associated inflammation

ILCs contribute to organ development and play important roles in the first line of antimicrobial defense, and can respond very quickly to signals or cytokines produced by other cells (99, 100). ILCs originate from the same common lymphoid progenitor as lymphocytes and their development relies strictly on the function of the transcription factor inhibitor of DNA binding-2 (Id2), other than Notch and IL-7 signaling (101, 102). However, ILCs lack some cell lineage markers associated with T and B lymphocytes, myeloid cells, and neutrophils, and express CD90, CD25, and IL-7 receptor α (CD127).

In the gut, ILCs are tissue-resident cells that are maintained and expanded locally under physiologic conditions, upon systemic perturbation of immune homeostasis, and during acute infection (103).

Three main subgroups of ILCs can be considered according to their developmental pathways, specific key transcription factors, and cytokine expression. The type 1 ILCs, including the natural killer (NK) cells and ILC1s, type 2 ILCs, and type 3 ILCs, which include lymphoid tissue-inducers (LTis), natural cytotoxicity receptor (NCR)+ ILC3s, which express NKp46 and T-bet, and double-negative ILC3s, which are CCR6 and NCR negative, and are the mixed precursors of CCR6+ LTi cells and NCR+ ILC3s. These 3 ILC subgroups share functional similarities with CD4+ Th1, Th2, and Th17 cells, respectively, whereas NK cells have similar roles to CD8+ cytotoxic T cells. ILC1s and NK cells express T-bet and produce IFNγ and TNFα. However, NK cells differ from ILC1s because their development is dependent on the transcription factor Eomesodermin (Eomes) and independent of Id2 (101).

ILC2s express high levels of GATA-3 and produce type 2 cytokines, while ILC3s express RORγt and produce IL-22 and IL-17 (100, 104–106). In addition to ILC1s and NCR+ ILC3s, T-bet can be induced in ILC2s (107, 108). Therefore, ILCs can secrete a variety of molecules by which they interact with many other cells within the mucosal environment, thus contributing to maintaining the mucosal homeostasis or perpetuating detrimental responses.

Changes in the number of ILCs, particularly ILC1s and ILC3s, have been documented in IBD and are associated with alterations of epithelial barrier integrity and production of cytokines. Particularly, the intraepithelial IFN-γ-producing CD127-expressing, NKp44-negative, c-kit-low ILC1 population is expanded in CD patients in response to IL-12 and IL-18 (109). This ILC1 population is not seen in the gut of alymphoid mice reconstituted with a human immune system, but it appears in the colons of mice with DSS-induced colitis (109). Despite this ILC subset is classified as ILC1, it also expresses low levels RORγt, raising the possibility that ILC1s can derive from RORγt-expressing ILC3s in inflamed tissues. Another ILC1-like subset, characterized by the expression of NKp44, NKp46, CD56, CD103, granzyme, and perforin, is located in the intestinal epithelial compartment and termed intraepithelial ILC1-like cells. In contrast to ILC1s, intraepithelial ILC1-like cells do not express IL-7Rα and do not rely on IL-15 for development and/or maintenance, even though they are functionally able to respond to IL-12 and IL-15 with enhanced secretion of IFN-γ (110). Intraepithelial ILC1s are increased in the inflamed gut of CD patients and are pathogenic in a murine model of colitis induced in Rag1-/- mice by anti-CD40 (110).

The contribution of ILC2s in IBD pathogenesis remains to be determined, though there is evidence that their frequency is elevated in the inflamed colons of UC patients and mice with the UC-like oxazolone-induced colitis (111, 112). It has also been shown that human IL-13-producing ILC2s can acquire the capacity to produce IFN-γ in response to IL-12, and ILC2s co-expressing IL-13 and IFN-γ are detectable in the inflamed gut of CD patients (108). Although these data support their inflammatory role, under specific circumstances, ILC2s may exert protective effects. For example, the number of IL-33-activated ILC2s producing the growth factor amphiregulin (AREG) increases in mice with acute colitis. Genetic loss of AREG exacerbates the ongoing colitis, while both the administration of exogenous AREG to mice and the transfer of ILC2s limit intestinal inflammation (113). The reason for such a discrepancy remains unclear, though it could reflect differences in the experimental models used.

ILC3s predominantly reside within the intestinal mucosal tissue, where they produce a series of effector molecules (e.g., RegIII β and RegIII γ, IL-17A, IL-22, and GM-CSF). The protective role of ILC3s is mainly mediated by IL-22. This is well evident in mice colonized with either segmented filamentous bacteria (SFB) or the invasive Escherichia coli (SFB) strain or treated with acetate and propionate, two ligands of free fatty acid receptor 2, in which production of IL-22 by ILC3s leads to intestinal epithelial cell repair and inhibition of the gut inflammation (114–116). The protective effects of IL-22 in epithelial cells are dependent on activation of AHR (117). AHR-null mice exhibit enhanced susceptibility to colitis and Citrobacter rodentium infection due to a reduction of ILC3s-derived IL-22 production in the intestine (118–120). Moreover, mice colonized with Lactobacillus reuteri D8 produce elevated levels of the metabolite indole-3-aldehyde, an AHR ligand, which enhances ILC3s-derived IL-22 production and attenuates DSS-colitis (121). Analysis of the mechanisms underlying the ILC3s-derived cytokine production suggests that distinct intracellular pathways are needed for the optimal activation of regulatory ILC3s in the gut. For example, RORγt-deficient mice do not exhibit defects in ILC3s-derived IL-22 production, which maintains gut health and prevents pathogen invasion. In contrast, such mice exhibit a diminished production of heparin-binding epidermal growth factor by activated ILC3s, which is needed for alleviating DSS-induced colitis (122). The production of effector cytokines by ILC3s is also regulated at the post-transcriptional level by a p38α-eIF6-Nsun2 axis, and defects in this pathway alter the production of protective cytokines, thus leading to increased susceptibility to colitis (123). A negative regulator of intestinal ILC3s-derived IL-22 production is SIRT6, a nicotinamide adenine dinucleotide-dependent deacetylase. Specific deletion of SIRT6 in ILC3s enhances IL-22 production without affecting the number of ILC3s, thus resulting in enhanced protection of DSS-induced colitis (124).

More recently, it was shown that the colons of mice with anti-CD40-induced inflammation contain RORγt-positive ILCs sharing markers of both ILC2s and ILC3s and producing IL-10. Deletion of the IL-10 gene specifically in such ILCs exacerbates both innate and adaptive immune-mediated experimental colitis (125).

Depending on the context where they are induced and activated, ILC3s can amplify rather than suppress intestinal mucosal inflammation. In the anti-CD40 induced colitis model, which is IL-23 dependent (126), ILC3s are a major source of GM-CSF, which is needed for these cells to move from cryptopatches to the intestinal tissue where they produce IL-22 and initiate an inflammatory immune cascade that results in intestinal inflammation (127). A somehow different scenario emerges from studies in Rag-deficient mice after infection with Helicobacter hepaticus, in which the development of IL-23-dependent colitis is associated with enhanced production of pathogenic IL-17A and IFN-γ by NKp46-negative ILC3s (128). Tbx21/Rag2 double-knockout mice develop spontaneously a UC-like intestinal inflammation, which is characterized by elevated production of IL-17A by ILC3s. Depletion of all ILCs or neutralization of IL-17A improves colitis in such a model (129).

The frequency of IL-17-producing ILC3s in the gut is positively regulated by the transmembrane protein neuropilin-1 (NRP1), the expression of which is increased in IBD tissue. Genetic deficiency of NRP1 reduces the frequency of intestinal ILC3s and impairs their ability to synthesize IL-17A, with the downstream effect of altering the composition of the microbiota and attenuating DSS-induced colitis (130). The proportion of ILC3s is increased in the inflamed colon of UC patients compared to healthy controls, and emerging evidence supports the view that the distribution and function of intestinal ILC3s are regulated by ferroptosis, a form of cell death characterized primarily by accumulation of reactive oxygen species and iron (131). Specifically, it was shown that induction of colitis is accompanied by upregulation of lipocalin-2 (LCN2) in ILC3s, particularly within the NKp46+ILC3 subpopulation. LCN2 triggers a p-p38-ATF4-xCT axis, which increases the expression of GPX4, thereby culminating in a block of ferroptosis and expansion of IL-22 and IL-17A-producing ILC3s (132). IL-23 and IL-1β trigger the IRE1α/XBP1 stress pathway in ILC3s through mitochondrial ROS production and, hence, enhance IL-17A and IL-22 production. The frequency of IRE1α/XBP1-expressing ILC3s is increased in the colons of mice with experimental colitis, as well as in the inflamed tissue of IBD patients, where it positively correlates with the response to treatment with ustekinumab, an IL-12/IL-23p40 blocker (133). Taken together, these results indicate that, in both acute and chronic models of innate immune-mediated colitis, ILC3s produce distinct patterns of cytokines, perhaps in response to specific environmental factors, thereby contributing to regulating gut pathology.

## MAIT cells in IBD

IBD patients exhibit significant changes in the frequency of mucosal-associated invariant T (MAIT) cells, a population of innate-like T cells that, unlike classical T cells, have a semi-invariant T cell receptor (TCR) composed of an invariant TCR Vα and Jα segment (TRAV1-2-TRAJ33/12/20), paired with a limited set of TCRβ chains, predominantly TRBV20 or TRBV6 in humans (134). Nearly two-thirds of human blood MAIT cells are CD4-CD8+ cells, 10- 15% are CD4/CD8 double negative, and the remaining are either CD4CD8 double positive or CD4+CD8- (135).

MAIT cells mediate rapid antimicrobial immune responses through either a TCR-dependent or TCR-independent mechanism. In the TCR-dependent pathway, MAIT cells recognize metabolites presented by the nonpolymorphic major histocompatibility complex class I-related protein (MR1), such as microbial metabolites derived from riboflavin biosynthesis. The most potent stimulatory MR1-binding ligands derived from riboflavin biosynthesis are 5-OP-RU [5-(2-oxopropylideneamino)-5-d-ribitylaminouracil] and 5-OE-RU [5-(2-oxoethylideneamino)-5-d-ribitylaminouracil] (134). This pathway activates specific transcription factors [e.g. T-box transcription factor TBX21 (T-bet), Eomes, RORγt, STAT3, and B lymphocyte–induced maturation protein 1 (BLIMP-1)], thus stimulating the production of various cytokines (e.g., IFN-γ, IL-17 and TNF), and cytotoxic molecules (e.g., perforin, granulysin, granzymes), which are needed in the host response to bacteria and fungi (136, 137) (**Figure 2**). The TCR-independent pathway is triggered by cytokines (e.g., IL-12 and IL-18) and enables MAIT cells to respond even in the absence of riboflavin metabolites and is crucial in the response to viruses and non-riboflavin-producing microbes (138). These two pathways often act either simultaneously or sequentially, thereby resulting in stronger effector responses.

In IBD, the frequency of MAIT cells is reduced in the blood and increased in the inflamed gut as compared to healthy donors (139), raising the possibility that, during the active phases of the disease, MAIT cells are recruited from the blood to inflamed tissues, as a result of the action of several factors (i.e. chemokines, chemokine receptors, and tissue adhesion molecules) (140–142).

Studies in the mouse Collaborative-Cross CC011/Unc strain, which spontaneously develops chronic colitis, have recently shown that MAIT cells accumulate in the colon. Such an expansion, which is driven by microbiota in an MR1-dependent manner, coincides with a loss of intestinal barrier permeability and induction of colonic inflammation. MAIT cells from colitic CC011 mice express IL-23R and produce high levels of IL-17A and IFNγ under the stimulus of IL-1 and IL-23, thus supporting the pathogenic role of MAIT cells. Consistently, deletion of the Traj33 gene, which is essential for MAIT development, attenuates colonic inflammation in this model (143). These data are in line with those produced in mice with oxazolone-induced colitis (113), in which both MR1 gene loss and orally administered isobutyl 6-formyl pterin, an antagonistic MR1 ligand, to wild-type mice attenuate colitis (144). In contrast, El Morr and colleagues showed that DSS-colitis led to a luminal expansion of riboflavin-producing bacteria, which was accompanied by enhanced production of MAIT ligands. MAIT ligands rapidly crossed the intestinal barrier and activated MAIT cells, thereby inducing tissue-repair genes. Specifically, MAIT cells spontaneously produced IFN-γ, which promoted mucus secretion by goblet cells, and IL-17A and IL-22, which stimulated the secretion of antimicrobial peptides and expression of tight-junction proteins in epithelial cells, thereby reinforcing the epithelial barrier. Finally, mice lacking MAIT cells were more susceptible to DSS-colitis and colitis-driven colorectal cancer (145). These observations support the view that MAIT cells are plastic and can adopt different programs during antigen recognition to cytokine challenges, and exert both inflammatory and anti-inflammatory roles (146).

## Ongoing inflammation in IBD is driven by various subsets of T cells

The IBD-associated mucosal lesions occur in areas that are massively infiltrated with several adaptive immune cells (i.e., T and B lymphocytes, memory T cells, T Follicular cells, stem-like CD4+T cells). These cells are mainly recruited from the blood circulation as a result of the action of several chemokines produced in the inflamed tissue. T cell trafficking to the intestine also relies on interactions between integrins (e.g., α4β7) and ligands expressed by endothelial cells (e.g., mucosal addressin cell adhesion molecule-1, intercellular adhesion molecule-1, vascular cell adhesion molecule-1) (**Figure 2**). The accumulation of lymphocytes in the intestinal mucosa has also been linked to the enhanced resistance of T cells to apoptotic stimuli, a phenomenon that appears to be more relevant in CD and dependent on the action of locally-produced cytokines (e.g., IL-6, IL-15, and IL-21) (147, 148). Mucosal T cells exhibit features of activated cells, express various transcription factors, and produce a vast array of effector cytokines. Specific subsets of T cells can be detected in the inflamed tissues of IBD patients, depending on the phases of the disease, the segment of the intestine involved, and the current treatment. Initially, it was believed that CD was a typical Th1-mediated pathology characterized by high production of IL-12 and IFN-γ (149), and elevated expression of Th1-associated transcription factors (i.e. Stat4 and T-bet) (150, 151), while UC was a Th2-mediated IL-4/IL-13-associated disease (152). More recent studies have shown that the inflamed gut of both CD and UC patients contains additional polarized Th cell subsets (e.g., Th17, Th9 cells, T follicular cells), which are supposed to contribute to expanding the pathological process (**Figure 2**). While the predominant accumulation of Th1 cells in CD and Th2 cells in UC reflects likely differences in the driving forces of the aberrant immune response in these two diseases, it remains unclear whether it could somewhat influence the course of the IBD as well as the responsiveness to current therapy. In this context, it is noteworthy that no benefit was documented in either patients with CD or patients with UC following treatment with drugs blocking the main effector cytokines produced by T cells, namely IFN-γ and IL-13, respectively (153, 154).

Additionally, in IBD mucosa, some subsets of T cells can co-express transcription factors that have been traditionally associated with the activation of different polarized T cells (e.g., co-expression of RORγt and Foxp3 or T-bet and RORγt). How naïve T cells polarize along specific subsets in the inflamed tissue of IBD patients is not fully understood, even though circumstantial evidence suggests that their commitment is largely influenced by cytokines produced within the inflammatory microenvironment. As pointed out above, in CD mucosa, there is high production of IL-12, the master inducer of Th1 cells, while IL-4 could play a major role in the differentiation of Th2 cells (155). In contrast, the induction of Th9 cells and Th17 (the latter expressing the transcription factors ROR-γt and RORα and producing various members of the IL-17 cytokine family, IL-21, and IL-22) is more complex and requires the concomitant presence/absence of various factors. For example, the differentiation of Th17 cells requires the concomitant action of TGF-β1 (the activity of which is also needed for the generation of peripheral Foxp3-expressing Tregs) and various cytokines that inhibit Foxp3 expression through the activation of the transcription factor Stat3 (i.e., IL-6, IL-21, IL-23) (156, 157).

Which cells produce the Th17-polarizing cytokines and which stimuli are needed for such production are unsolved questions, though it is known that resident microbiota and microbiota metabolites (e.g., short-chain fatty acids, polyamines, secondary bile acids, and indole derivatives) can influence the differentiation and stabilization of Th17 cells in the gut. Indeed, germ-free mice have reduced numbers of T cells in the gut but their colonization with commensal bacteria restores the number and function of both Th17 cells and Tregs (158).

Notably, the transfer of IBD microbiotas into germ-free mice increases the numbers of intestinal Th17 cells while it decreases the frequency of Tregs, and exacerbates T cell-dependent colitis (159), indicating that IBD microbiotas contain strains that preferentially induce Th17 cell responses and exacerbate the ongoing inflammation (115). This is in line with the demonstration that some specific microbial populations, such as SFB, or bacterial components (i.e. SFB-derived flagellins), are powerful inducers of intestinal Th17 cells (160).

Molecularly, SFB enhances the epithelial cell-derived production of serum amyloid A (SAA), the latter being able to substitute for TGF-β1 in the induction of Th17 cells (161, 162).

The accumulation of Th17 cells in the gut can also be induced by Propionibacterium, Prevotella, and Lactobacillus casei (163–165). Altogether, these data confirm and expand on previous studies showing that, in IBD and colitic mice, there is a loss of T cell tolerance towards normal components of the gut microbiota (166–169).

Analysis of T cells isolated from human colon samples has revealed the presence of some CD4+ and CD8+ T lymphocyte subsets characterized by gene expression profiles (i.e., TCF1 and BCL6) resembling stem-like progenitors and clonally related to pathogenic TH17 cells. These cells, termed stem-like T cells, are increased in the inflamed colon of UC patients and are pathogenic in the T cell-transfer model of colitis (170). It is thus conceivable that stem-like T cells might be the source for pathogenic Th17 cells (and perhaps pathogenic CD8+ T cells).

Hegazy and colleagues showed that microbiota-reactive CD4+ T cells are normal constituents of the human gut, even though such cells are more abundant in IBD tissue and produce predominantly IL-17A as compared with responses of T cells from blood or intestinal samples of normal controls (171). Such cells represent an example of CD4+ memory T (TRM) cells, which are generated from effector T cells, and have a long lifespan and the ability to self-proliferate and produce effector molecules, including inflammatory cytokines (**Figure 2**). In addition to CD4+ TRM cells, the gut contains CD8+ TRM cells, which are mainly located in the epithelial layer. TRM cells express surface markers, such as CD49a, CD69, and CD103, but lack chemokine receptor 7 and L-selectin (CD62L), which ensure the long-term presence of TRM cells within tissues (172). It is, however, noteworthy that, like effector T cells, TRM cells are heterogeneous, and some subtypes do not express CD69 and CD103 (173).

Several cytokines regulate the differentiation of intestinal TRM cells. TGF-β1 appears to be a master inducer of TRM differentiation and retention in the gut due to its ability to induce CD103 and downregulate KLF2 (174, 175). Differentiation of TRM cells is also promoted by TNF-α, IL-6, and IL-33, all of which cooperate with TGF-β1, while IL-12 and IFN-β are inhibitors (176). Many researchers have assessed the distribution and role of TRM cells in IBD. In an elegant study, Neurath’s group showed that CD69+CD103+ TRM cells accumulated in the mucosa of patients with IBD and produced high levels of inflammatory cytokines (i.e., IFN-γ, IL-13, IL-17A, and TNF-α), and the presence of CD4+CD69+CD103+ TRM cells was predictive of IBD exacerbations. Furthermore, these authors documented attenuated colitis in both mice with functional impairment of TRM cells due to a double knockout of the TRM-cell-associated transcription factors Hobit and Blimp-1 and following depletion of TRM cells (177). Along the same line is the demonstration that induction of DSS colitis is associated with an increase in the number of T-cell immunoreceptor with immunoglobulin and ITIM (TIGIT)-expressing CD4 + TRM cells producing IL-17A and IFN-γ and TIGIT deficiency inhibits IL-17A production by such cells, resulting in attenuation of colitis (178). Bishu and colleagues confirmed the abundance of CD4+ TRM cells in CD and showed that such cells are major producers of TNF-α and IL-17A, a finding that was at least in part dependent on the expression of PRDM1 (179). By assessing the expression of CD103 and KLRG1, two receptors of E-cadherin expressed by intestinal epithelial cells, Bottois and colleagues documented high numbers of CD103+ CD8+ T cells in the CD mucosa and showed that such cells express high levels of Ki67 and NKG2A, indicating that they are more responsive to TCR triggering. In contrast, KLRG1+ CD8+ T cells have increased cytotoxic and proliferative potential (179).

Further analysis showed that a subset of CD103+CD4+TRM cells expressing CD161 and CCR5 are specific for CD but not UC. These cells exert cytotoxic activity and produce high levels of inflammatory cytokines (180). Other authors have documented a reduced number of both CD103+CD4+ and CD103+CD8+T-cell subsets in CD, probably reflecting both differences in the IBD patient backgrounds and heterogeneity of TRM cells (181).

By single-cell RNA and antigen receptor sequencing, Boland and colleagues documented different states of differentiation of CD8+ TRM cells in the human colon, with a predominance of inflammatory cells expressing T-bet and Eomes in UC mucosa (182).

Less is known about the contribution of Th9 cells, a subset of Th cells that produce IL-9 but not Th1, Th2, and Th17-related cytokines, in IBD. Th9 cells are abundant in the inflamed mucosa of UC patients, and the presence of IL-9 in such a condition is associated with significant changes in the expression of proteins that regulate epithelial barrier integrity (183, 184). Since, IL-9 belongs to the common γ chain family of cytokines, and like other cytokines of this family can control the function of many immune (e.g., T cells, B cells, ILCs, mast cells) other than epithelial cells (185), Th9 cells likely exert pathogenic cells by modulating the function of many mucosal cell types.

In UC, Th9 cells exhibit high expression of αEβ7, which is supposed to mediate the homing of Th9 cells to the intestine and retention. Consequently, treatment of UC patients with Etrolizumab, a blocker of the β7 subunit, reduces the colonic numbers of Th9 cells (186).

The frequency of Th9 cells is increased in mice with various forms of IBD-like colitis (i.e., DSS- and TNBS-colitis and T cell transfer colitis), and neutralization of IL-9 in such models attenuates the ongoing inflammation (184, 187).

Induction of IL-9 in Th9 cells is mediated by PU.1, an ETS family transcription factor, which directly binds to the IL-9 gene locus (188). The factors that promote Th9 cell differentiation in IBD are not fully characterized. Initial studies showed that the differentiation of Th9 cells is promoted by the concurrent action of TGF-β1 and IL-4 or IL-4 and IL-1β (189, 190). Recent studies in the ileitis-prone SAMP mice have convincingly shown that Th9 cell induction can be promoted by the death receptor 3 (DR3), a member of the TNFR superfamily 25, which is preferentially expressed on activated T cells and acts as the functional receptor for TNF-like cytokine 1A (191). IL-36 family members can also promote Th9 cell differentiation and inhibit Foxp3-expressing Tregs induction, as shown by studies in mice deficient in IL-36 signaling, in which the increased numbers of colonic Tregs, and reduced frequencies of Th1 and Th9 cells were associated with protection from T cell-derived intestinal inflammation (192, 193). Another positive regulator of Th9 cell development is IL-33, an epithelial cell-derived cytokine, which is upregulated in UC patients (194, 195).

Together, these findings indicate that the active phases of IBD patients are marked by the presence of various subsets of effector CD4+ T cells, which can secrete a vast array of cytokines and chemokines, thus contributing to expanding the mucosal immune response.

## Regulatory T cells

As mentioned above, Tregs are a subset of Foxp3-expressing T cells involved in the induction and maintenance of immune homeostasis and tolerance (196). Although the majority of Tregs are generated in the thymus (tTregs) under the stimulus of multiple signals (e.g., TCR activation, CD28 costimulation, cytokines), they can also be differentiated in the periphery (pTregs), including the gut, from mature CD4+ T cells through a process mainly involving TGF-β1 (56, 197). Induction of pTregs in the gut relies on the intestinal microbiota because both germ-free and wild-type mice receiving a broad-spectrum antibiotic cocktail have a diminished frequency of colonic Tregs and, hence, become more susceptible to colitis (198, 199). These findings are in line with the demonstration that either non-pathogenic Clostridia strains and short-chain fatty acids generated from fiber-fermenting bacteria stimulate TGF-β1-mediated pTreg differentiation with the downstream effect of limiting colitis (200, 201). The development and regulatory functions of Tregs rely on Foxp3, and in patients with Foxp3 mutations, Tregs are absent in number or function, and this defect is associated with the development of intestinal inflammation (202).

Tregs are a major source of TGF-β1 and a large body of evidence indicates that this cytokine plays a key role in the Tregs-induced suppressive action (203, 204). However, not all the regulatory effects of Tregs would seem to rely on TGF-β1. For example, TGF-β signalling is dispensable for Tregs-induced suppression of Th1 cell differentiation, while it is needed for Tregs to suppress Th17 cells and regulate responses in the gastrointestinal tract, as a result of the promoting effect of TGF-β1 on molecules that retain Tregs in the colon (i.e., CD103, GPR15) (205). The reader is directed towards recent reviews on the role of Tregs in maintaining intestinal homeostasis and dampening intestinal inflammatory responses (206–208). In this context, it is, however, noteworthy that the frequency of Tregs in the inflamed IBD tissue is increased (209), raising the possibility that the inability of Tregs to control the IBD-associated inflammation is secondary to their reduced function rather than an insufficient number. Indeed, effector T cells isolated from the inflamed gut of CD patients are resistant to Tregs-mediated immunosuppression, a finding that has been associated with Smad7-dependent block of TGF-β1 function (210). Additionally, Foxp3-expressing Tregs are plastic and they can acquire the functional properties of effector T cells. For instance, Th17-like Tregs are enriched in the inflamed colon of IBD patients, express higher Th17-related cytokines and lower immunosuppressive cytokines compared with typical Tregs (211). Finally, Tregs isolated from patients with CD express lower levels of α4β7 than Treg cells isolated from control individuals, a defect that hampers Tregs recruitment and suppressive function (212).

## B-cell and plasma cell responses in IBD

B cells and IgA+ and IgG+ plasma cells accumulate in the inflamed tissue of IBD patients (**Figure 2**), but their exact contribution to the pathogenesis of these disorders is not yet well-known. Treatment of UC patients with rituximab, a monoclonal antibody depleting CD20-positive B cells, had no significant effect on inducing remission in steroid-unresponsive, moderately active UC, supporting the view that B cell responses are not pathogenic in this disease (213). In UC, there is also production of IgG antibodies against tropomyosin 5, an antigen expressed by epithelial cells in the colon and other sites (e.g. eyes and biliary tract) (214, 215) as well as IgG antibodies anti-neutrophil cytoplasmic antibody (ANCA) and against the colonic epithelial integrin αvβ6, the latter being correlated with disease severity and associated with adverse UC-related outcomes (216–219).

In contrast, CD patients have increased levels of IgG and IgA against Saccharomyces cervisiae, flagellin, and E. coli (220–222).

In the inflamed tissue of IBD patients, there is a high number of granzyme B-expressing CD19(+) and IgA(+) cells, which co-express CD27 and CD38, and can kill *in vitro* intestinal epithelial cells, raising the possibility that such cells could contribute to IBD-associated epithelial damage (223). By single-cell RNA sequencing, single-cell IgH gene sequencing, and protein-level validation of blood and mucosal samples, Uzzan and colleagues documented in the inflamed UC colon the existence of an auto-reactive plasma cell clone targeting αvβ6 integrin and identified a subset of intestinal CXCL13-expressing T Follicular cells that were associated with the pathogenic B cell response. Such changes in intestinal humoral immunity are reflected in circulation by the expansion of gut-homing plasmablasts that correlate with disease activity and predict the development of complications (224). In UC mucosa, there is induction of anti-commensal IgG, and commensal-IgG immune complexes target FcγR-expressing macrophages, thereby stimulating ROS-dependent production of IL-1β, type 17 immunity, and eventually exacerbating DSS-colitis (225).

On the other hand, there is evidence that some subsets of B cells/plasma cells could be anti-inflammatory in IBD. For example, CD11b-positive B cells expressing high levels of CD21, CD23, IgD, and IgA accumulate in the inflamed colon of mice with DSS-colitis and UC patients, and their adoptive transfer to DSS-treated mice attenuates colitis through a mechanism, which relies on CD11b and appears to be independent on the ability of such cells to influence the gut microbiota (226).

Moreover, in the peripheral blood and colon of UC patients, there is a reduced frequency of CD24 ^high^ CD38 ^high^ and CD5-expressing regulatory B cells (Bregs), which correlates with the clinical activity of the disease and inflammatory biomarkers (227).

## Conclusions

In recent years, the advent of sophisticated molecular techniques has advanced our knowledge of the pathogenesis of IBD. There is now sufficient evidence to believe that many innate and adaptive immune cells make a valid contribution to the IBD-associated pathological process, even though the exact sequence of molecular events that drive the tissue-damaging immune response remains to be determined, as well as whether the main immune alterations described in this article occur simultaneously or sequentially in individual patients. Nonetheless, there is a large consensus in believing that, during the active phases of the disease, the various immune cell types secrete a vast array of effector cytokines, which can target both immune cells and non-immune cells (e.g., epithelial cells, stromal cells), thus triggering signals that amplify the mucosal inflammation. Indeed, clinical trials and real-life studies show that monoclonal antibodies targeting TNF, the IL-12/IL-23p40 subunit, or the specific IL-23p19 subunit are effective in IBD. Consistently, blocking the recruitment of immune cells into the gut of IBD patients with antibodies targeting integrins is helpful. The fact that such antibodies promote the resolution/attenuation of the clinical manifestations and endoscopic/histological signs in both UC and CD supports the view that the targeted cytokine/integrin plays an active role in the mucosa-damaging immune response in both diseases (228–232). However, not all patients respond to the available therapeutic compounds, highlighting the heterogeneity of the IBD population. Indeed, patients with similar symptoms and endoscopic alterations may exhibit different immune-mediated transcriptomic signatures in the colon (233) and, even in the same patient, it is possible to document changes in the mucosal immune response and production of effector cytokines over time, which could account for the responsiveness to biologics (234). During the exacerbations of both CD and UC, multiple cytokines produced by distinct subsets of effector immune cells can contribute to triggering inflammatory signals that culminate in the same tissue damage. This has facilitated new therapeutic strategies based on the use of either a single drug inhibiting intracellular pathways activated by many cytokines (e.g., JAK/STAT inhibitors) or two different compounds with complementary actions (235).

## Future directions

Despite the promising results obtained with these novel approaches, additional studies are needed to identify better candidates for specific therapies. Some demographic, clinical, and molecular features seem to predict responsiveness to biologics and small molecules (236–238). Nonetheless, we do not yet know whether such features correspond to the expression/function of specific mediators in the damaged intestinal mucosa. Several biomarkers have been explored to predict response to therapy in IBD, aiming to support a more personalized approach to treatment (94). The development of biomarkers to predict therapeutic response or disease severity, although not yet applicable in clinical practice, has only been made possible by a deeper understanding of the molecular events occurring in the affected tissue of IBD patients. Among these, elevated baseline levels of oncostatin M and its receptor have emerged as one of the most consistent predictors of non-response to anti-TNF therapy. Moreover, genetic and pharmacogenetic studies have identified variants such as IL23R polymorphisms, which correlate with infliximab response, and HLA-DQA1*05, which is associated with increased risk of immunogenicity. Similarly, transcriptomic and proteomic analyses have highlighted the predictive potential of specific mucosal gene expression profiles, though reproducibility across independent cohorts has been challenging. The gut microbiome has also been implicated in treatment outcomes, with higher baseline microbial diversity and the presence of short-chain fatty acid–producing species being associated with better response to anti-TNF agents, vedolizumab, and ustekinumab (237).

Advanced endoscopic techniques have enabled *in vivo* or ex vivo quantification of mucosal target cell populations. For example, a higher density of membrane-bound TNF-expressing cells in the intestinal mucosa has been linked to infliximab responsiveness, while increased expression of α4β7 integrin has predicted better outcomes with vedolizumab (94). Despite this growing body of evidence, no single biomarker has yet demonstrated sufficient accuracy, reproducibility, and feasibility to guide clinical decision-making. The integration of multi-omics data, combining genetic, microbial, transcriptomic, proteomic, and cellular insights, represents the most promising strategy to stratify patients and implement true precision medicine in IBD (239).
